# Understanding the Threats Posed by Non-Native Species: Public vs. Conservation Managers

**DOI:** 10.1371/journal.pone.0053200

**Published:** 2013-01-16

**Authors:** Rodolphe E. Gozlan, Dean Burnard, Demetra Andreou, J. Robert Britton

**Affiliations:** Centre for Conservation Ecology and Environmental Sciences, School of Applied Sciences, Bournemouth University, Fern Barrow, Poole, Dorset, United Kingdom; Swansea University, United Kingdom

## Abstract

Public perception is a key factor influencing current conservation policy. Therefore, it is important to determine the influence of the public, end-users and scientists on the prioritisation of conservation issues and the direct implications for policy makers. Here, we assessed public attitudes and the perception of conservation managers to five non-native species in the UK, with these supplemented by those of an ecosystem user, freshwater anglers. We found that threat perception was not influenced by the volume of scientific research or by the actual threats posed by the specific non-native species. Media interest also reflected public perception and vice versa. Anglers were most concerned with perceived threats to their recreational activities but their concerns did not correspond to the greatest demonstrated ecological threat. The perception of conservation managers was an amalgamation of public and angler opinions but was mismatched to quantified ecological risks of the species. As this suggests that invasive species management in the UK is vulnerable to a knowledge gap, researchers must consider the intrinsic characteristics of their study species to determine whether raising public perception will be effective. The case study of the topmouth gudgeon *Pseudorasbora parva* reveals that media pressure and political debate has greater capacity to ignite policy changes and impact studies on non-native species than scientific evidence alone.

## Introduction

Conservation managers recognise that public support can be critical to the success of the activities they undertake [1]. Public opinions concerning one conservation issue, managing non-native species in the environment, are generally determined by the perceived ecological benefits, the financial costs and ethical issues associated with the management actions [2], [3]. As the management of non-native species often includes eradication programs, it can be ethically challenging [4], especially when it involves the culling of species that the public find appealing [5]. Consequently, public opinion is frequently used to underpin the management of non-native species with, for example, conservation managers in Australia and New Zealand using public surveys to help them understand the current attitudes of people to non-native species generally and the likely reaction of the public to proposed management schemes specifically [6], [7]. Where public attitudes are not considered in management programs, the consequences can be far reaching, such as in California, USA, where an eradication of pike *Esox lucius* proceeded with inadequate public consultation and resulted in lawsuits being taken out against the regulatory authorities responsible [8].

Given this importance of public opinion and perception in ensuring conservation schemes meet their desired objectives, researchers must disseminate their results effectively into the public domain if they wish to influence conservation and ecological policies, and assist in the formulation of management programs [2]. Moreover, researchers are increasingly under pressure to ensure their work provides social, economic and/or cultural impacts, i.e. have benefits beyond academia [9]. Successful dissemination of research into the public consciousness is, however, dependent on external factors such as generating and then maintaining media interest [10]. Without this, the importance of the work may be arguably considered irrelevant to the wider sphere of conservation as it fails to enter the public domain sufficiently to influence public perceptions and policy. Notwithstanding, it tends to be easier to raise awareness of conservation issues when the focal species has intrinsic characteristics that are appealing, i.e. the species is likely to elicit an immediate and emotive reaction in most people and so they receive more media attention [1]. It is these species, such as the giant panda *Ailuropoda melanoleuca*, that tend to be used to front conservation programs [11]; whilst this increases their profile and appeal still further, it does not necessarily translate into successful conservation for either the target species or for biodiversity on a broader scale. [12].

When public opinion is used to help prioritise conservation efforts, there is a risk that some species will inherently receive more attention than others through media bias, with a danger that much of the underlying scientific research is then disregarded [13]. Indeed, where research fails to be integrated into the conservation planning process, management failures are likely due to research-implication gaps [14]. Failure to incorporate research into policy-making has already been identified as detrimental in some conservation contexts, including within ecological restoration [14] and endangered species protection [15], [16]. For example, Knight et al. (2008) demonstrated that numerous tools developed to guide conservation assessment typically are not applied in the selection of nature reserves [14] and Linklater (2003) revealed that during a period of rapid decline in rhinoceros populations, policy was not determined by ecological studies that would otherwise have increased the success of conservation efforts [17].

Given the apparent importance of public opinion in underpinning the management of non-native species [1], [8] and for prioritising those species for actions [11], the aim of this study was to examine the relationship between the research outputs of a range of non-native species and the corresponding perception on those species by the public, conservation management planners and the exploiters of one ecosystem type affected by non-native species (freshwater anglers). The research questions were: (1) Are there higher levels of public perception for certain invasive species regardless of the ecological risk they pose? (2) Which factors influence public attitudes to the ecological risks posed by invasive species? and (3) Are conservation priorities of non-native species being overlooked by managers, i.e. is the management of invasive species vulnerable to knowledge gaps? The spatial area used to complete the research was the UK and a case study of the invasive fish topmouth gudgeon *Pseudorasbora parva* [18]–[28] is used to provide an example of how research can successfully influence conservation management.

## Results

### Return Rate and Demographic Statistics

Of the 409 people invited to participate in the street survey, 187 (46%) returned questionnaires. The social–demographic profile of the respondents closely matched that of the total population when compared with the 2011 census [29]. There was a higher proportion of female respondents (56% female, 44% male) but this was not significantly different from the gender proportions in the population (chi square test, *X* ^2^ = 0.001, d.f. = 1, *P* = 0.988. The age demographics of the survey did not differ significantly from that of the total population (*X* ^2^ = 0.596, d.f. = 5, *P* = 0.982).

### Perception of General Threats Posed by Non-native Species

**Figure 1:**
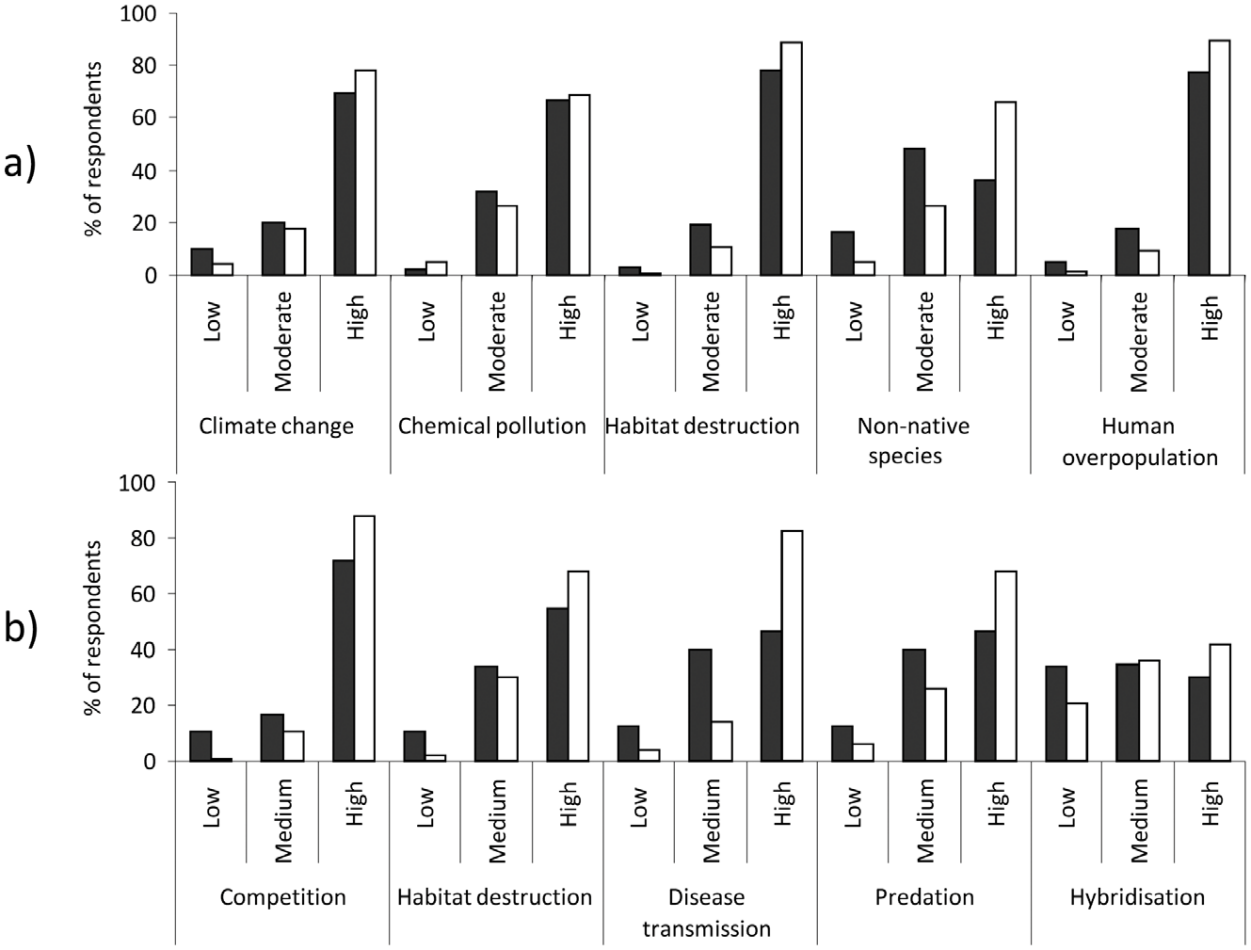
Public and conservaton managers’ threat perception concerning conservation issues. Public (black; n = 186) and conservation manager (white; n = 132) threat perception (%) for a) anthropogenic driven conservation issues and b) non-native species introductions.

Conservation managers (n = 132) perceived three anthropogenic disturbances as significantly greater conservation threats than perceived by public respondents (n = 187): habitat destruction (Mann Whitney *U* test, *z* = 2.71, *P* = 0.007) human overpopulation (*z* = 4.00, *P*<0.001) and non-native species introductions (*z* = 6.05, *P*<0.001). A larger proportion of conservation managers than public respondents regarded these issues as high threat anthropogenic disturbances (Figure 1a). Perceptions especially varied between these two groups on non-native species introduction, with 16% of the public respondents classing these as a low ecological threat compared to only 5% of conservation managers. There were no significant differences in threat perception between conservation managers and the public for climate change (*z* = 1.90, *P* = 0.06) and chemical pollution (*z* = 0.12, *P* = 0.904).

**Figure 2:**
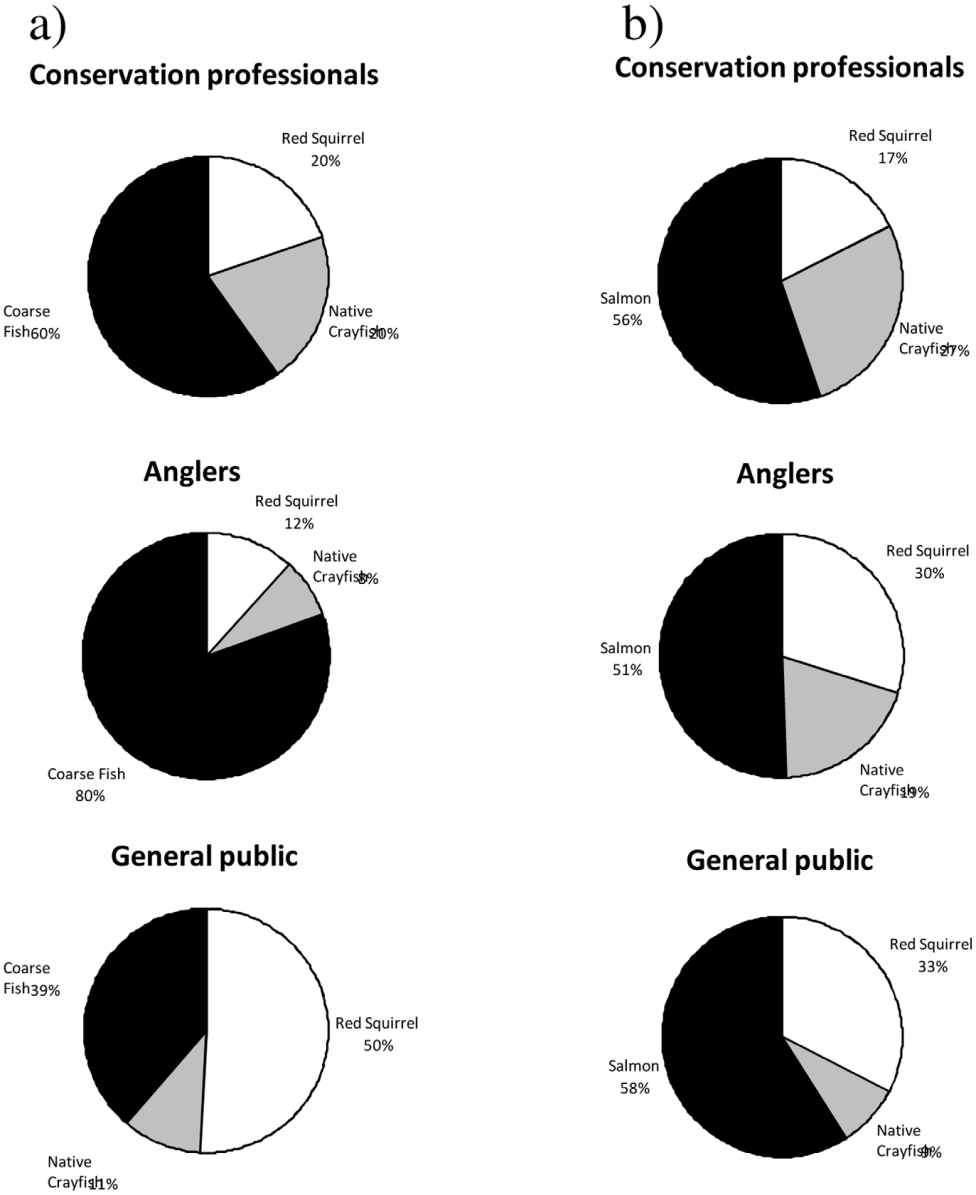
Evaluation of UK conservation concerns. Conservation managers (n = 132), public respondents (n = 186) and anglers (n = 103) were asked to select one species from a range, (a) red squirrel, native crayfish or coarse fish, (b) red squirrel, native crayfish or salmon, for which a disease to, would cause the most ecological concern.

Conservation managers perceived the following issues associated with non-native species as being significantly greater conservation threats than the public: competition (*z* = 5.92, *P*<0.001), habitat destruction (*z* = 3.34, *P*<0.001), disease transmission (*z* = 6.27, *P*<0.001), predation (*z* = 4.16, *P*<0.001) and hybridisation (*z* = 2.70, *P*<0.001). Perceptions especially varied between the two groups on disease transmission; 47% of public respondents thought this was a high conservation risk compared to 83% of conservation managers (Figure 1b). The public perceived a disease to red squirrels as a higher concern than diseases to course fish or native crayfish, but conservation managers and anglers were most concerned about impacts to coarse fish (Figure 2). Disease threats to salmon raised more concern than threats to red squirrels or native crayfish in both public and conservation manager respondents (Figure 2).

### Knowledge and Threat Perception of Specific Non-native Species

**Figure 3:**
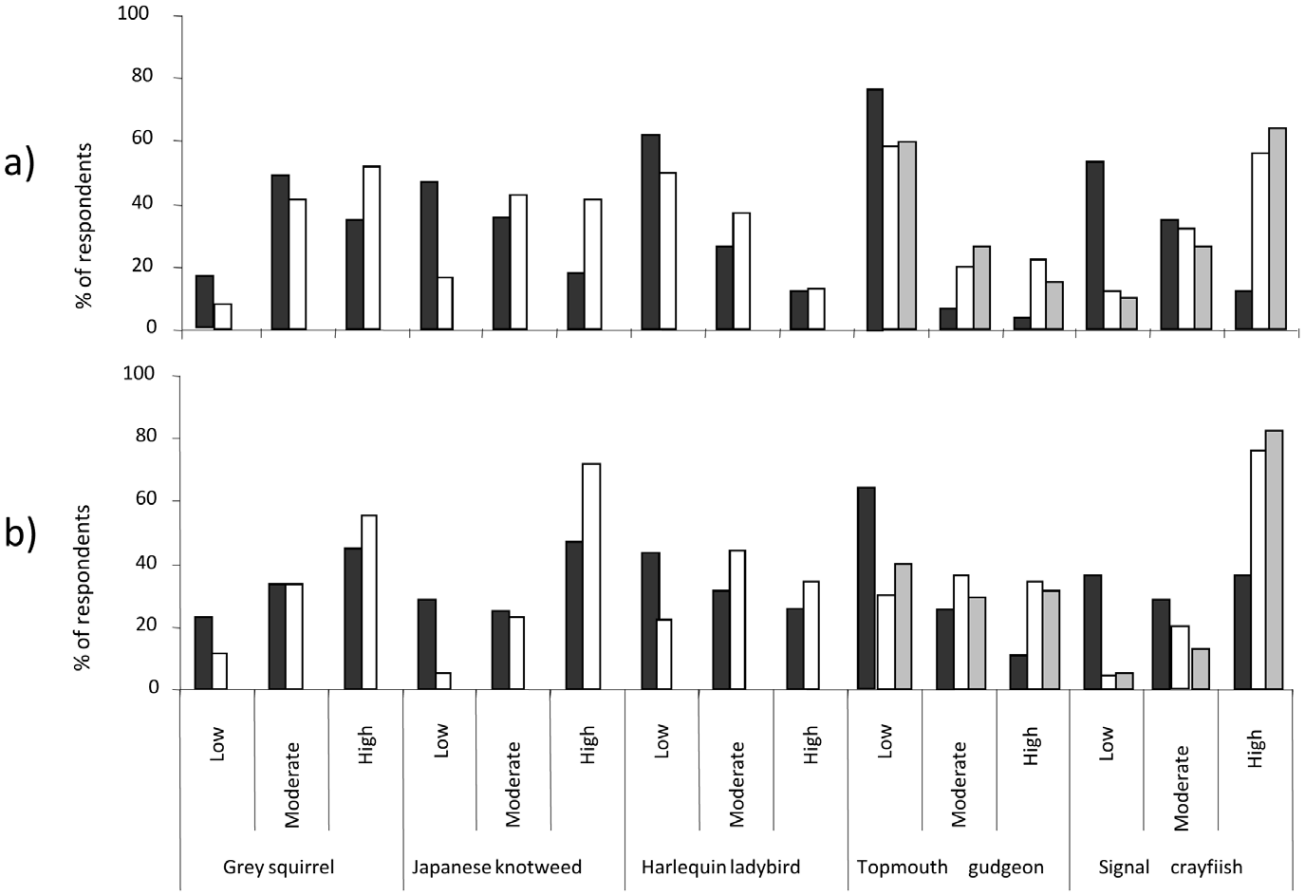
Evaluation of knowledge and threat perception concerning non-native species in the UK. (a) Knowledge (%) and (b) threat perception (%) of non-native species in the UK by the public (black; n = 186), conservation managers (white; n = 132) and anglers (grey; n = 103).

Conservation managers (n = 132) perceived a significantly greater knowledge than the public (n = 187) for all the non-native species surveyed: grey squirrel, (Mann Whitney *U* test *z* = 2.0, *P* = 0.46), Japanese knotweed, (*z* = 5.16, *P*<0.001), signal crayfish, (*z* = 8.12, *P*<0.001), harlequin ladybird, (*z* = 3.29, *P*<0.001) and topmouth gudgeon (*z* = 6.82, *P*<0.001). The majority of the public perceived a moderate (49%) or high level (35%) of knowledge concerning grey squirrels, with their awareness of the other non-native species being considerably lower and especially limited for harlequin ladybird and topmouth gudgeon (Figure 3a). A larger proportion of anglers surveyed (n = 103) perceived a high level of knowledge concerning signal crayfish (64%) than topmouth gudgeon (15%) (Figure 3a). A large proportion of conservation managers perceived a high level of knowledge concerning signal crayfish (56%) and grey squirrels (52%) but knowledge concerning topmouth gudgeon was more limited, with 58% of conservation managers perceiving a low level of knowledge (Figure 3a).

Conservation managers perceived a significantly greater ecological risk than public respondents for all the non-native species sampled: grey squirrel (,*z* = 2.86, *P* = 0.004), Japanese knotweed (*z* = 6.34, *P*<0.001), signal crayfish (*z* = 9.09, *P*<0.001), harlequin ladybird (*z* = 2.41, *P* = 0.016) and topmouth gudgeon, (*z* = 7.80, *P*<0.001). A large proportion of public respondents perceived Japanese knotweed (46%), grey squirrel (44%) and signal crayfish (36%) as high ecological risks (Figure 3b) but only 11% and 22% perceived the same level of risk for the topmouth gudgeon and harlequin ladybird respectively (Figure 3b). The large majority of anglers (83%) perceived signal crayfish as a high risk but topmouth gudgeon was regarded as a low risk by more respondents (40%) than those which regarded the species as a moderate (29%) or high risk (31%). While a greater proportion of conservation managers than public respondents or anglers perceived topmouth gudgeon as a high risk species, 40% regarded the species as a low ecological risk (Figure 3b) and perceived grey squirrels (*z* = 6.47, *P*<0.001), signal crayfish (*z* = 8.12, *P*<0.001) and Japanese knotweed *z* = 5.16, *P*<0.001) as significantly greater ecological risks.

### Media Output

**Figure 4:**
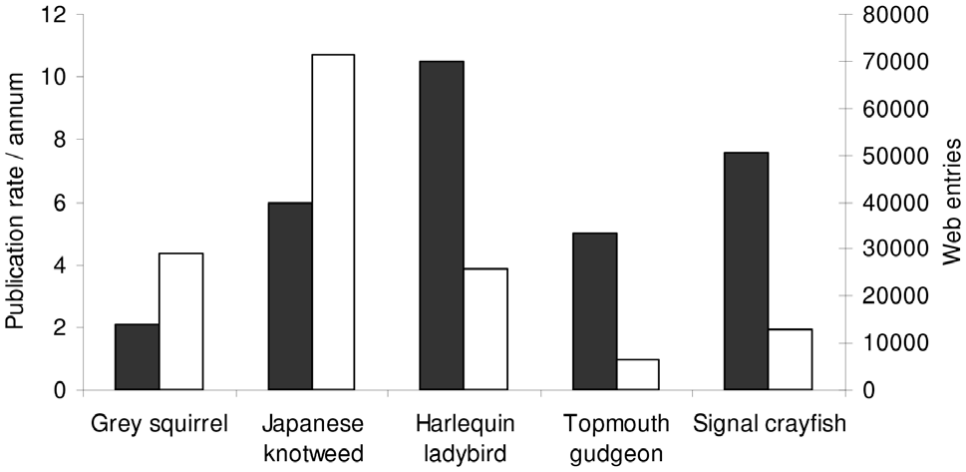
Correlation between scientific research and media concerning invasive species resident in the UK. Publication rate per annum (black) and web entries (number of internet hits) (white) concerning non-native species in the UK.

Internet representation (most to least) was determined as: Japanese knotweed, grey squirrel, harlequin ladybird, signal crayfish and topmouth gudgeon (Figure 4). Representation for topmouth gudgeon was approximately 10% of the coverage devoted to Japanese knotweed. The signal crayfish received approximately twice the amount of web coverage than topmouth gudgeon (Figure 4).

### Research Outputs

Productivity (most to least) per annum was determined as: harlequin ladybird, signal crayfish, Japanese knotweed, topmouth gudgeon and grey squirrel (Figure 4). Publication rate concerning the ecological impacts posed by the grey squirrel was particularly low (2 per annum).

### Ecological Risks of the 5 Non-native Species

Mean scores were determined as: topmouth gudgeon = 22 (S.E. = 0.4), Japanese knotweed = 19 (S.E. = 0.4), harlequin ladybird = 13 (S.E. 0.7), signal crayfish = 12 (S.E. = 1.1) and grey squirrel = 8 (S.E. = 0.7). Based on these scores, two of the five species, the topmouth gudgeon and Japanese knotweed are considered to pose serious ecological risks.

## Discussion

This study attempted to provide a wider view of public and conservation managers opinions on invasive species than had been previously attempted in the UK. We also attempted to determine the extent to which opinions are matched by scientific evidence and provide insights to how perceptions are reflected by external sources such as the media. All questionnaire based studies have limitations in respect to how representative of the general population they are [30]. We used a random street survey sampling process to minimise sampling bias but recognise that the people which responded may have different personality traits from the people that did not and consequently may also have different opinions, therefore, some sampling bias may still exist. As respondents were unaware of the subject matter, non response bias and self selection bias were both minimised and because there are broad similarities between the social demographics of respondents and the general population and the return rate was high (46%), the data can be used to assess public attitudes to invasive species.

### Are there Higher Levels of Public Perception for Certain Invasive Species Regardless of the Ecological Risk they Pose?

This study clearly demonstrated that public awareness (i.e. knowledge) of non-native species does not correlate with their actual ecological risks. In fact, public awareness of the species associated with the highest quantified risk, the topmouth gudgeon, was extremely low. In comparison, public awareness of the grey squirrel, the species with the lowest associated ecological risk of the five species tested, was very high. While previous studies have identified a taxonomical prejudice [1] especially in favour of mammals [31], these results demonstrate a clear bias towards knowledge on terrestrial species compared with aquatic. The three terrestrial species were perceived to be better understood than the two aquatic representatives, which, may demonstrate that personal observation is a primary contributing factor influencing the degree of perception towards invasive species. Further research is required to determine whether the public perceive established familiar invasive species well but have less awareness of emerging ecological threats.

### Which Factors Influence Public Attitudes to the Ecological Risks Posed by Invasive Species?

Our results suggest that, in isolation, scientific research has a negligible impact on public opinion. There was no correlation between public awareness or threat perception of individual non-native species with the intensity of their research outputs. Species subjected to intense research, such as the harlequin ladybird [32], [33] and signal crayfish [34], [35], still did not have high levels of public awareness. The public also appear to value native species, which have intrinsic characteristics that they find appealing (e.g. red squirrels) or are linked to a food source/recreational activity (e.g. salmon). It follows that raising awareness of invasive species which impact on organisms with no perceived public value will be inherently difficult for researchers to achieve.

Our results also demonstrated a strong correlation between the volume of media output for a particular species and public awareness, with high media volume reflected in a high level of public perception and vice versa. Although, not surprising, this correlation most likely represents a cycle where articles are produced on subjects that editors recognise to be familiar to the public and are likely to be read, but disproportionate media exposure to these same species reinforces public perception and conservation managers. Many conservation issues have benefited from increased media interest [36] but our results may suggest that this approach will not be successful when the study species are unfamiliar to the public. Exceptions may occur when emerging threats have intrinsic characteristics or even names, which are perceived to be interesting. The case of the ‘killer shrimp’ *Dikerogammarus villosus*, a recent invader to the UK [37], has recently attracted a high level of media interest and illustrates how sensationalism can cause media interest regardless of the specific threat posed to biodiversity.

### Is Invasive Species Management Vulnerable to Knowledge Gaps?

More concerning is the perceptions of conservation managers to non-native species that were closely aligned with those of the public and anglers, rather than matching the scientific research and quantified ecological risk. Awareness of the harlequin ladybird, for example, is low despite the species being subject to intense research in recent years [32], [33]. The topmouth gudgeon is identified to be one of the most successful and potentially damaging invasive species of the last thirty years [18], but is regarded by conservation managers to cause the least ecological risk of the five tested species. As conservation managers appear ill informed of the ecological risks posed by invasive species, our study suggests that invasive species management in the UK is highly vulnerable to knowledge gaps. Better communication between scientists conducting primary research and conservation managers implementing policy is required as well as a strong scientific underpinning for the prioritisation of conservation efforts regarding non-native species management.

**Figure 5:**
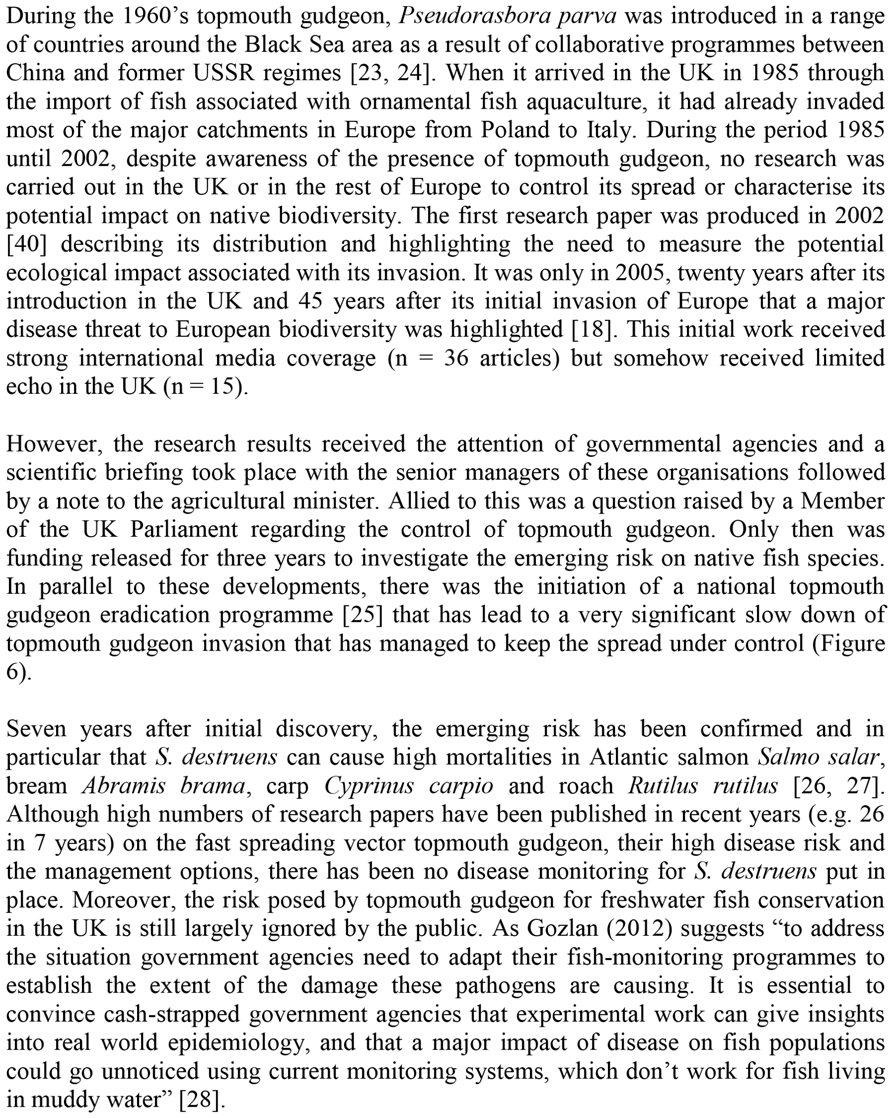
Influencing Invasive Species Management Policy. The case study of topmouth gudgeon *Pseudorasbora parva* in the UK.

**Figure 6:**
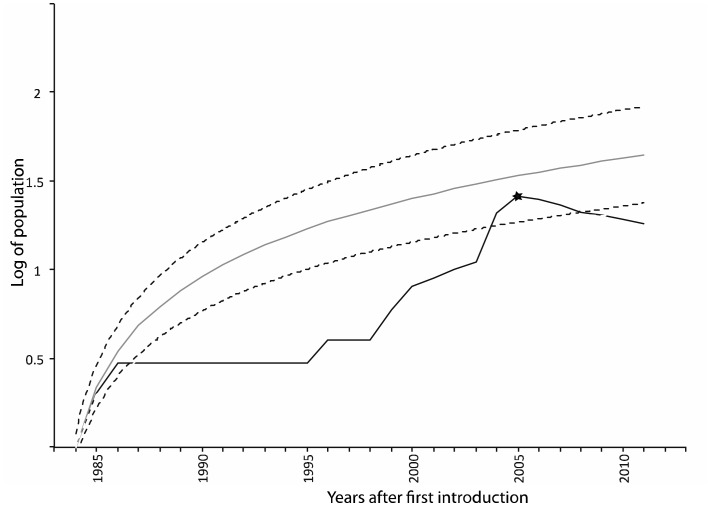
Topmouth gudgeon *Pseudorasbora parva* invasion. Predicted populations (grey line; y = 0.417ln(x) +0.2461; R^2^ = 0.97) based on European invasion data [23] and 95% confidence interval (dash line). Effective topmouth gudgeon populations detected in the UK during the same period (black line) with the star indicating the start of the national eradication programme.

As the opinions of conservation managers do not appear to reflect scientific research, a pertinent question is: how can research influence policy in species whose threat is underestimated by conservation managers? In examples where control of spread and risk management is extremely time sensitive we suggest a direct approach similar to that used to influence policy concerning the topmouth gudgeon (Figure 5). Here, with the case of topmouth gudgeon, it is clear that despite strong scientific evidence [18]–[28], only media pressure and questions in parliament has ignited impact studies and re-active management (i.e. eradication programme). The other lesson learnt is that aggressive eradication management has been very successful in containing topmouth gudgeon’s invasion (Figure 6) and that combined with an early warning system it would significantly reduce the risk of future invasion in the UK.

While this study aimed to identify public and conservation managers’ perceptions of invasive species and highlight potential pathways for their opinions, further research is required to explore the mechanisms underlying knowledge sharing and the formation of perceptions among these groups. It was not possible in this study, for example, to accurately identify the casual factors influencing the perceptions of conservation managers but it is expected to be similar to the public. Therefore, our results suggest that researchers should not assume that by publishing their work in academic journals they will subsequently influence opinions and policies. A more transparent rationalisation of conservation priorities (i.e. which species should receive primary attention) as well as a better use of science findings to underpin management strategies is needed. Discussions between the two stakeholders should focus on developing objective risk assessment processes, which can quickly be used to determine risk on which control programmes can be based on. In the absence of effective collaboration, direct appeals to governmental stakeholders may be employed and researchers need to consider the species in question before deciding on the best action.

## Materials and Methods

To answer the three research questions, data were collected and analysed through questionnaires (Q1 to 3), media outputs on non-native species (Q2), scientific literature (Q2, 3) and risk assessment of non-native species (Q1, 3).

### Ethics Statement

This project was approved by Bournemouth University Research Ethics Committee. A Research Ethics checklist was prepared, submitted and approved to document this process and is held on file by Bournemouth University. Participants gave their written consent for participation in the study. The following statement was included in the questionnaire: 'BU is undertaking an opinion survey related to public view on non-native species. The outcome of the survey will be published in a peer reviewed journal but remain anonymous with exception of age and sex of participants. Would you consent to participate in this study and answer questions related to this subject?' The outcome was recorded as an approval tick box as part of the survey form. The consent procedure was approved by Bournemouth University Research Ethics Committee.

### Perception Questionnaire

To determine current attitudes to invasive species, a questionnaire approach was used. The questionnaire (see supplementary material) was completed by conservation managers, the public and freshwater anglers. Face to face surveys with the public were conducted at Bournemouth city centre (n = 84) Cardiff city centre (n = 64), and central London (South bank) (n = 38) on the 11^th^, 13^th^ and 14^th^ July 2011 respectively. Anglers completed 103 questionnaires, with the majority (87) obtained from surveys posted on the following internet based forums on 29^th^ June 2011: Talk Angling (www.talkangling.co.uk; n = 31), Anglers Net (www.anglersnet.co.uk; n = 48); Carp forum (www.carpforum.co.uk; n = 24). The remaining 16 questionnaires were obtained from the public surveys from respondents who answered positively to the question ‘do you go fishing?’ Conservation managers also completed an online questionnaire with 132 completed from the following organisations: CEFAS (http://cefas.defra.gov.uk/; n = 24), Natural England (http://www.naturalengland.org.uk/; n = 43) and the Environment Agency (www.environment-agency.gov.uk; n = 15), with 41 returned from numerous other environmental charities and organisations, including the National Trust (n = 1) and Freshwater Biological Association (n = 1).

The questionnaire had two principal sections: (i) perception of general threats posed by non-native species; and (ii) knowledge and threat perception of specific non-native species. Within the questionnaire, non-native species were defined as ‘an animal or plant introduced (i.e. by human action) outside its natural past or present distribution: that has the ability to spread and potential to cause damage to the environment, the economy, our health and the way we live’.

- 1 **Perception of general threats posed by non-native species.** The questionnaire asked respondents for their perception of the conservation threats posed by anthropogenic disturbances and of the general threats associated with non-native species introductions. To aid identification of the causal factors contributing to threat perception, respondents were also asked to select species from two lists for which a disease would cause the most ecological concern: 1 = red squirrel *Sciurus vulgaris*, white-clawed crayfish *Austropotamobius pallipes* or ‘coarse’ (caught for sport) fish, 2 = red squirrel, white-clawed crayfish or Atlantic salmon *Salmo salar*).
- 2 **Knowledge and threat perception of specific non-native species.** In these questions, respondents were asked for their level of knowledge and threat perception of five non-native species: the grey squirrel *Sciurus carolinensis* introduced to the UK in 1876 [38], harlequin ladybird *Harmonia axyridis* (2004 [39], topmouth gudgeon *Pseudorasbora parva* (1985) [40], signal crayfish *Pacifastacus leniusculus* (1976) [41] and Japanese knotweed *Fallopia japonica* (1825) [42]. The five species were selected on the basis of their representation of different taxonomic groups in different ecosystems and their known invasiveness in the UK [43].

Other than the lists of species, each question was answered using a five-point rating scale with the knowledge options of 1: extensive, 2: much, 3: some, 4: little, and 5: none. The data were then analysed between sample groups for each question using a pair-wise Mann Whitney *U* test on the 5 point rating scale. The data were then grouped as low (none and little combined), moderate (some) and high (much and extensive combined) for use in qualitative comparisons between sample groups. The analysis primarily focused on the differences in knowledge between conservation managers and the public, with this supplemented by angler knowledge. The rationale for this was that anglers represented a narrow sector of ecosystem users who, when compared with the other two groups, would have a biased knowledge base towards freshwater species.

### Quantifying Media Output for each Non-native Species via Web Presence

To assist determination of the factors which influence public attitudes to the ecological risks posed by invasive species (Q2), their media coverage was quantified through their internet presence. Consequently, internet searches for each non-native species using the search terms ‘invasive species’ and ‘non-native species’ were conducted using three popular UK search engines: Google (www.google.co.uk), Yahoo (http://uk.yahoo.com) and Bing (http://www.bing.com) on the 25^th^ March 2011, according to the guidelines for systematic review [44]. Search terms were separated by Boolean operators (Table 1). Regarding analysis, the total number of internet ‘hits’ for each of the three search engines was summed for each non-native species and the mean calculated to enable comparisons between the species.

**Table 1:** Search strings allocated to non-native species.

Species	Search strings
Grey Squirrel *Sciurus carolinensis*	(‘Invasive species’ OR ‘non native’) AND (‘grey squirrel’ OR ‘gray squirrel’ OR ‘*Sciurus carolinensis*’)
Harlequin ladybird *Harmonia axyridis*	(‘Invasive species’ OR ‘non native’) AND (‘harlequin ladybird’ OR ‘Asian lady beetle’ OR ‘Japanese ladybug’ OR ‘*Harmonia axyridis* **’**)
Topmouth gudgeon *Pseudorasbora parva*	(‘Invasive species’ OR ‘non native’) AND (‘topmouth gudgeon’ OR ‘stone moroko’ OR ‘*Pseudorasbora parva*’)
Signal crayfish *Pacifastacus leniusculus*	(‘Invasive species’ OR ‘non native’) AND (‘signal crayfish’ OR ‘american crayfish’ OR ‘*Pacifastacus leniusculus*’)
Japanese knotweed *Fallopia japonica*	(‘Invasive species’ OR ‘non native’) AND (‘Japanese knotweed’ OR ‘*fallopia japonica*’)

Search terms used to conduct internet searches (using Google, Yahoo and Bing) and locate scientific publications using Web of Knowledge.

### Quantifying Research Outputs for Each Non-native Species

To determine whether the intensity of invasive species research influences public and conservation managers’ perceptions of invasive species (Q1,2), the number of peered review research publications concerning each of the five non-natives was found using the search terms ‘invasive species’ and ‘non-native species’ separated by Boolean operators (Table 1) on the Web of Knowledge database (http://wok.mimas.ac.uk). The total number of publications concerning each of the five non-natives was used for comparisons between species.

### Quantifying the Ecological Risks of the 5 Non-native Species

To determine whether the perceptions of non-native species reflected their actual ecological risks (Q1, 3), their risks were quantified using an adapted Fish Invasiveness Scoring Kit (FISK) [45] for risk assessment of both terrestrial and aquatic species. Six questions out of 49 from the original tool, specific to fish invasions (11, 20, 21, 22, 28 & 29) were omitted for analysis. It uses a scoring system to assess species based on the basis of their biogeography and history, biology and ecology [46]. Higher scores indicate an increased risk of the species being invasive following an introduction, and calibration has revealed species with scores ≥19 to be those that pose the greatest risk [46]. Although originally conceived to pre-screen species either proposed for introduction or likely to be introduced [45]; this risk assessment tool has been successfully incorporated into post-introduction assessments [46]. The mean of two independent assessments (completed by two different people) was used to determine the ecological risk of each of the five non-native species.

## Supporting Information

Text S1 Perception questionnaire used in this study.
(DOC)

Dataset S1 Data obtained from perception questionnaire.
(XLS)
