# Supplementary material for: Understanding the Threats Posed by Non-Native Species: Public vs. Conservation Managers
Source: PLoS One. 2013 Jan 16;8(1):e53200. doi: 10.1371/journal.pone.0053200 (PMC3547005; doi:10.1371/journal.pone.0053200)
Supplement: Text S1 — Perception questionnaire used in this study. (DOC) [file pone.0053200.s001.doc]

ATTITUDES TO NON-NATIVE SPECIES IN THE UK

For the purposes of this survey 'Non-native species' refers to an animal or plant introduced (i.e. by human action) outside its natural past or present distribution; that has the ability to spread and potential to cause damage to the environment, the economy, our health and the way we live.

At Bournemouth University our research on non-native species underpins policy and management used to protect biodiversity. Your replies will help us better understand how scientific research modifies general attitudes towards non-native species.

Thank you for taking the time to complete this questionnaire. There are no correct answers; it is your opinion that we are interested in. The survey is anonymous.

**PART 1. ABOUT NON-NATIVE SPECIES**

1. In 2004 Colonel Robert J. Pratt, of the U.S. Army wrote, “Despite America’s status and strength, the United States was tragically vulnerable to attack not from conventional weapons, but by unconventional asymmetric means. Today the homeland is vulnerable to a different type of asymmetric attack, a biological attack from invasive species.”

Do you agree or disagree that non-native species could represent a threat of national importance?

Strongly agree Agree Neither agree nor disagree Disagree Strongly disagree

**□ □** **□ □** **□**

2. How do you think the UK government should prioritise spending in the following areas?

Most Very Important Not Very Not

Important Important Important Important

Healthcare **□** **□** **□ □ □**

Education **□** **□** **□ □ □**

Economic **□** **□** **□ □ □**

Development

Defence **□** **□** **□ □ □**

The Environment **□** **□** **□ □ □**

3. Please indicate your perception of the threat that the following environmental issues pose:

Extensive Much Some Little None

Climate change **□** **□** **□ □ □**

Chemical pollution **□** **□** **□ □ □**

Habitat destruction **□** **□** **□ □ □**

Non-native species **□** **□** **□ □ □**

Human overpopulation **□** **□** **□ □ □**

4. Do you have any prior knowledge on the subject of non-native species?

Extensive Much Some Little None

**□ □** **□ □** **□**

5. Please indicate your level of knowledge on each of the following five species:

Extensive Much Some Little None

Grey Squirrel **□** **□** **□ □ □**

Japanese Knotweed **□** **□** **□ □ □**

American Crayfish **□** **□** **□ □ □**

Harlequin ladybird **□** **□** **□ □ □**

Topmouth gudgeon **□** **□** **□ □ □**

6. Please indicate where you have heard about non-native species:

**□** Television **□** Newspapers **□** Internet

**□** Scientific journals  **□** Radio **□** Magazines

**□** Word of mouth **□** I haven’t heard about such issues

**□** Other (please specify)____________

7. Please indicate your perception of the threat the following five species pose:

Extensive Much Some Little None

Grey Squirrel **□** **□** **□ □ □**

Japanese Knotweed **□** **□** **□ □ □**

American Crayfish **□** **□** **□ □ □**

Harlequin ladybird **□** **□** **□ □ □**

Topmouth gudgeon **□** **□** **□ □ □**

8. Please indicate your perception of the level of threat posed by these issues associated with non-native species:

Extensive Much Some Little None

Competition **□** **□** **□ □ □**

Habitat destruction **□** **□** **□ □ □**

Disease transmission **□** **□** **□ □ □**

Predation **□** **□** **□ □ □**

Hybridisation **□** **□** **□ □ □**

(inter-breeding with native species)

9. If a disease existed that could obliterate either red squirrels or native crayfish or native coarse fish (eg roach/tench/bream), which would cause you most concern?

**□**Red squirrels  **□**Native crayfish  **□**Coarse fish

10. If a disease existed that could obliterate either red squirrels or native crayfish or salmon, which would cause you most concern?

**□**Red squirrels  **□**Native crayfish  **□** Salmon

11. Which of the following should be the primary reason for controlling non-native species:

**□**Recreational use of the environment **□**Economic cost of damage

**□**Loss of ecological function **□**Intrinsic value of wildlife **□**Other (please specify): ____________

12. Prior to this survey had you ever heard about research by Bournemouth University scientists on the non-native fish, Topmouth Gudgeon?

**□**Yes  **□**No  **□** Not sure

**PART 2. ABOUT YOU**

13. What is your gender?

**□** Male **□** Female

14. What is your age?

**□** 18-34 **□** 35-44 **□** 45-54

**□** 55-64 **□** 65-74 **□** 75+

15. Please indicate the highest level qualifications you have (tick one):

**□** ‘O’ level, GCSE, *or equivalent*

**□**  ‘A’ Level, AS Level, *or equivalent*

**□** NVQ, BTEC, City and Guilds, *or equivalent*

**□** First degree (e.g. BA, BSc)

**□** Higher degree (e.g. MA, MSc, PhD, PGCE, post-graduate certificates/diplomas)

**□** Other qualifications (please specify) __________________________________________

**□** No qualifications

16. What is the first half of your postcode (Home address)?

E.g. BH12________________________________________________________________

17. Do you go fishing?

**□**No **□**Yes (Coarse fishing) **□**Yes (Sea angling) **□**Yes (Salmon and trout)

18. What is your occupation?

________________________________________________________________________

19. Finally, do you have any other comments you’d like to make about the subject of this survey?

**__________________________________________________________________________________________________________________________________________________________________________________________________________________________________________________________________________________________________________________________________________________________________________________________________________________________________________________________________________________________________________Thank you**
